# Simultaneous Determination of Purpurin, Munjistin and Mollugin in Rat Plasma by Ultra High Performance Liquid Chromatography-Tandem Mass Spectrometry: Application to a Pharmacokinetic Study after Oral Administration of *Rubia cordifolia* L. Extract

**DOI:** 10.3390/molecules21060717

**Published:** 2016-06-01

**Authors:** Mingjie Gao, Jing Yang, Zhibin Wang, Bingyou Yang, Haixue Kuang, Lu Liu, Liqian Wang, Chunjuan Yang

**Affiliations:** 1 Department of Pharmaceutical Analysis and Analytical Chemistry, College of Pharmacy, Harbin Medical University, 157 Baojian Road, Nangang District, Harbin 150081, Heilongjang, China; gaomingjie8888@163.com (M.G.); a1534064875@163.com (L.L.); wangliqian93@163.com (L.W.); 2 Analytical Department, Johnson & Johnson, 199 Grandview Road, Skillman, NJ 08558, USA; jjsprite09@gmail.com; 3 Key Laboratory of Chinese Materia Medica (Ministry of Education), Heilongjiang University of Chinese Medicine, 24 Heping Road, Xiangfang District, Harbin 150040, Heilongjang, China; wzbmailbox@126.com (Z.W.); ybywater@163.com (B.Y.); hxkuang@yahoo.com (H.K.)

**Keywords:** UHPLC-MS/MS, *Rubia cordifolia* L., purpurin, munjistin, mollugin, pharmacokinetics

## Abstract

A specific, simple, sensitive Ultra High Performance Liquid Chromatography-tandem Mass Spectrometry (UHPLC-MS/MS) method has been developed and validated for the simultaneous determination and pharmacokinetic study of purpurin, munjistin, and mollugin in rat plasma. Chromatographic separation was carried out using a C_18_ column (ACQUITY UPLC^®^ HSS T3, 1.8 μm, 2.1 × 100 mm) with gradient elution. The compounds were detected on a 6430 triple-quadrupole tandem MS with an electrospray ionization (ESI) interface using multiple reaction monitoring (MRM) in positive ionization mode. The samples were prepared by a liquid-liquid extraction (LLE) method with ethyl acetate after being spiked with an internal standard (bifendate). The current UHPLC-MS/MS assay was validated for its linearity, intra-day and inter-day precisions, accuracy, extraction recovery, matrix effect and stability in different conditions. The method was linear for all analytes over the investigated range with all determined correlation coefficients exceeding 0.9900. The intra-day and inter-day precisions were in the range of 4.21% to 14.84%, and the relative errors of accuracies were in the range of −14.05% to 14.75%. The mean recoveries and matrix effects of purpurin, munjistin, and mollugin were higher than 78.87% and 92.56%, repectively. After oral administration of 0.82 g/kg of *Rubia cordifolia* extract, the maximum plasma concentrations (*C*_max_) were 70.10 ± 11.78 ng/mL for purpurin, 26.09 ± 6.6 ng/mL for munjistin, and 52.10 ± 6.71 ng/mL for mollugin. The time for maximal concentration (*T*_max_) was 1.61 ± 0.24 h for purpurin, 2.58 ± 0.19 h for munjistin, and 1.99 ± 0.21 h for mollugin. The established method was further applied to a pharmacokinetic study of purpurin, munjistin, and mollugin in rat plasma. It was concluded from the pharmacokinetic parameters that the three analytes showed a process of slow absorption and metabolism after oral administration of *R. cordifolia* extract to rats.

## 1. Introduction

*Rubia cordifolia* L., belonging to the genus Rubia (Rubiaceae), is a type of perennial herb, distributed widely around the world, including the western and northern parts of Europe, the Mediterranean coast, temperate regions of Asia and Africa, as well as the regions from Mexico to Tropical America [1]. The genus Rubia comprises about 70 species, among which a total of 36 species and two varieties have been reported in China. The dried roots and rhizomes are referred to in Traditional Chinese Medicine as *Rubiae Radix et Rhizoma*, and is widely used for the treatment of menoxenia, rheumatism, contusions, hemorrhages, chronic bronchitis and trauma, and is officially listed in the 2015 edition of the Chinese Pharmacopoeia [2]. Previous phytochemical investigations have shown that this plant is a source of cyclic hexapeptides, anthraquinones, and arborinane-type triterpenoids [3]. *R. cordifolia* exhibited cytotoxicity in various cancer models, including leukemia, melanoma, colon cancer, laryngeal squamous cell carcinoma, as well as lung cancer [4,5,6,7,8]. In addition, *R. cordifolia* has a long history in skin care and treatment, and has been used in disorders of the urinary tract [9].

**Figure 1:**
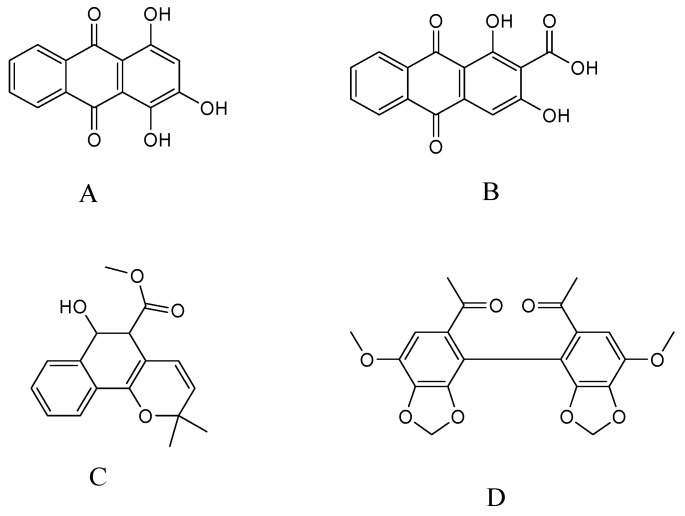
Chemical structures of purpurin (**A**); munjistin (**B**); mollugin (**C**); and I.S. (**D**).

In our previous investigations, three compounds: purpurin, munjistin, and mollugin (Figure 1) were isolated and identified from the 70% ethanol extract of *R. cordifolia*. Purpurin and mollugin are the main indicator compounds for this composition in the Chinese Pharmacopoeia [2]. Purpurin and mollugin have been reported to possess antioxidant and enzyme inhibition activities. In particular, purpurin showed stronger antioxidant and better enzyme inhibitory effects than mollugin [10]. Mollugin is a naturally benzochromene reputed for its anti-carcinogenic and anti-viral activities [11]. In addition, mollugin might be a broad action inhibitor, inhibiting LPS-induced inflammatory responses and suppressing the inflammatory responses in lipopolysaccharide (LPS)-stimulated RAW 264.7 macrophages [12]. Hydroxyanthraquinones have been reported to be the predominant antioxidant phenolic constituents in the roots of *R. cordifolia* [13]. Purpurin is a novel specific inhibitor of adipocyte-derived leucine aminopeptidase (A-LAP) and could be developed as a new anti-angiogenic agent [14]. Studies have demonstrated the potent *in vitro* anti-fungal activities of purpurin in terms of inhibiting six *Candida* species [15].

Various analytical methods, including ultra-fast liquid chromatography [16], high-performance liquid chromatography [17,18,19], and ultrasound-assisted ionic liquid-reversed phase liquid chromatography [20] have been used in the *in vitro* qualitative or quantitative analysis of munjistin, purpurin, and mollugin. However, there is no report on the simultaneous determination of the three compounds using a single method. LC coupled with triple-quadrupole MS method has been widely used in pharmacokinetic studies because its multiple reaction monitoring (MRM) technique provides high sensitivity for quantification [21,22,23,24]. In this paper, we have developed for the first time a tested sensitive and selective UHPLC-MS/MS method to simultaneously determine munjistin, purpurin, and mollugin in rat plasma. The validated method has then been successfully applied to the pharmacokinetic study of the three components for the first time. The results of this pharmacokinetic study could provide some useful references for the further pharmacological study of *R. cordifolia*.

## 2. Results and Discussion

### 2.1. Optimization of the UHPLC-MS/MS Analysis

The first step in our method development was to select a suitable ionization mode in order to obtain the precursor and product ions of the analytes and I.S. In our previous investigations, anthraquinone compounds have been shown to be the major active substances in the roots of *R. cordifolia*. Anthraquinones are polyhydroxy compounds, easily releasing protons, and in theory their response signals are higher in negative than positive ionization mode [25]. Mollugin is a naturally benzochromene, and its chemical structure is very different from that of purpurin and munjistin, which are anthraquinones. Our first priority was to employ negative ionization mode to obtain the precursor and product ions of the analytes, however the response of mollugin was very bad (*S*/*N* < 10) and the peak area of mollugin in negative mode was only a few hundred counts when its concentration was 1 μg/mL. Moreover, tailing of the three analytes obviously occurred when the response signal for purpurin and munjistin was good. We changed the mobile phase using methanol-water (0.1% HCl) and methanol-water (5 mM ammonium acetate) to improve the peak shape of the analytes and the tailing problem was greatly improved for purpurin and munjistin. 

**Figure 2:**
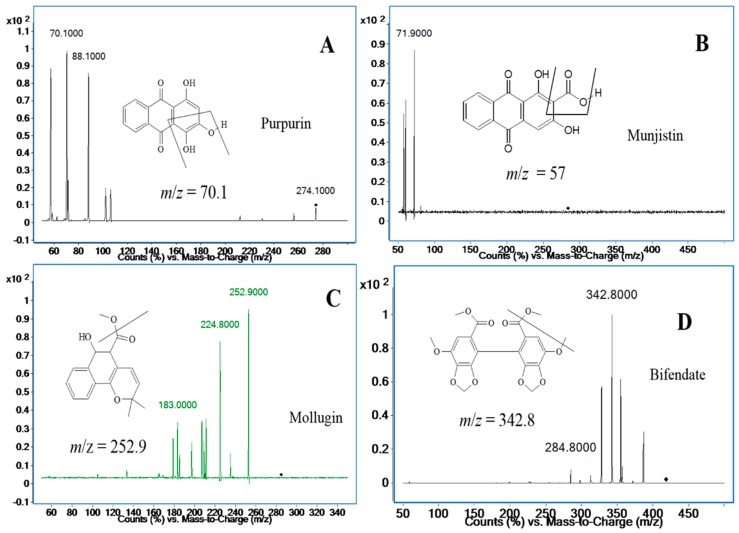
Product ion mass spectra of purpurin (**A**); munjistin (**B**); mollugin (**C**); and bifendate (I.S.) (**D**).

Unfortunately, the response signal for mollugin was worse than before, so it was decided to use positive ionization mode to detect mollugin, and the response of mollugin appeared more stable and stronger in this mode than that in negative mode. Applying both ionization modes to simultaneously determine the three analytes might be a good choice in terms of sensitivity and separation, however, it would seriously damage the mass spectrometer capillary, so positive mode has to be applied to all three compounds, even though the positive ion mode was not the best choice for anthraquinones. After adjusting the mobile phase, satisfactory responses of the three analytes were achieved. The ion pairs of the three analytes and the I.S. in positive ion mode are shown in Figure 2. The analytes yielded protonated molecular ions ([M + H]^+^ or [M + NH_4_]^+^) as the base peak. The parameters of the fragmentor and collision energies were optimized in order to obtain the richest relative abundance of precursor and product ions. MS/MS transitions and energy parameters of all the compounds are listed in Table 1. The selection of a proper internal standard is another key step, which could improve the method performance. Bifendate was chosen as the I.S., as it exhibited a stable response, effective separation, good reproducibility and extraction efficiency. 

Chromatographic conditions were optimized to improve the peak shape, increase the sensitivity and shorten the run time for simultaneous analysis of the three components. Several mobile phase systems were tested to identify the optimal mobile phase in order to produce the best response, sensitivity and separation efficiency. The mobile phase systems of methanol-water and acetonitrile-water at various ratios were tested, and different buffers including ammonium acetate (2 and 5 mM), acetic acid (0.1% and 0.2%), and formic acid (0.1% and 0.4%) were evaluated. Finally, a mobile phase of methanol and 5 mM ammonium acetate in water was selected as the best solvent mixture. Satisfactory separation was achieved in 8.5 min by gradient elution using an ACQUITY UPLC^®^ HSS T3 C_18_ column (1.8 μm, 2.1 × 100 mm, Waters, Milford, MA, USA) at 40 °C with a flow rate of 0.4 mL/min. There were no endogenous components in rat plasma interfering with the analytes when the mobile phase mentioned above was used. Comparing the elution profiles of blank plasma spiked with the three analytes and the I.S. with that of real plasma sample, additional peaks around mollugin were observed in the real plasma sample chromatograms. This phenomenon may be caused by other isomers or compounds absorbed in the blood, which have the same molecular mass of the precursor and product ions after one or more cleavage [26,27]. So we optimized the liquid phase conditions to adjust the retention time so as to obtain the suitable resolution of the three analytes and eliminate the crosstalk among the three compounds.

### 2.2. Sample Preparation

To establish the sample preparation procedure, both liquid-liquid extraction (LLE) using ethyl acetate, acetone, chloroform, or dichloromethane, and protein precipitation by acetonitrile and methanol have been evaluated. The response of the three analytes was higher with liquid-liquid extraction by ethyl acetate than using protein precipitation and the analytes could be extracted effectively in real blood plasma. In comparison with other solvents, ethyl acetate has many advantages that it could provide clean, simple and reproducible extraction, and it is friendly to the environment. In addition, ethyl acetate can easily volatilize under the nitrogen gas at 40 °C, which could improve the throughput of the experiments.

### 2.3. Method Validation

#### 2.3.1. Specificity and Selectivity

**Figure 3:**
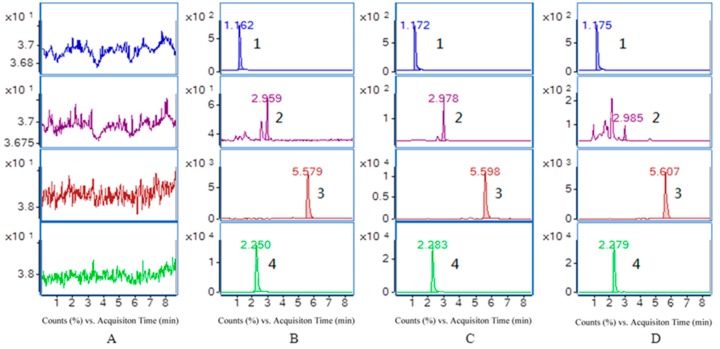
Representative MRM chromatograms of I.S. (1), mollugin (2), munjistin (3) and purpurin (4) in rat plasma (**A**) a blank sample (without the three analytes and I.S.); (**B**) LLOQ sample (three analytes and I.S.); (**C**) QC sample (three analytes and I.S.) and (**D**) a rat sample taken 2 h after administration of 0.82 g/kg *R. cordifolia*.

The selectivity of the endogenous plasma matrix was evaluated using blank plasma from six rats. The retention times of purpurin, munjistin, mollugin and the I.S. were 2.250, 5.579, 2.959, and 1.162 min, respectively. Typical chromatograms obtained from a blank, a spiked plasma sample with the analytes and the I.S. and a plasma sample collected 2.0 h after oral administration of *R. cordifolia* are shown in Figure 3.

All the analytes and I.S. peaks were detected with good resolution and symmetrical peak shapes, and there were no interference peaks from endogenous substances at this elution times of the analytes and the I.S. The analytes could be easily different from the rat plasma matrix and quantitatively determined at the LLOQ level. Therefore, it can be concluded that the present method is selective for determination of three components in rat plasma.

#### 2.3.2. Linearity and Lower Limits of Quantification

The calibration curves of purpurin, munjistin, and mollugin in rat plasma provided reliable responses at the concentration range of 0.264–264 ng/mL, 0.515–515 ng/mL, and 2.45–2450 ng/mL, respectively. The best linear fit and least-square residuals for the calibration curve were achieved with a weighting factor of 1/*x*^2^. All correlation coefficients exceeded 0.9900 (0.9927 for purpurin, 0.9979 for munjistin, and 0.9939 for mollugin). The LLOQ of purpurin, munjistin, mollugin were 0.264, 0.515, and 2.45 ng/mL, respectively. The regression equation, correlation coefficients and linear ranges for the three analytes are shown in Table 2.

#### 2.3.3. Precision and Accuracy

The results of the intra-day and inter-day precision and accuracy are listed in Table 3. At each QC level, the intra- and inter-day precisions (RSD) of the three analytes were less than 14.99%, and the accuracy ranged from −14.05% to 14.75%. Thus, the developed method was precise and accurate.

#### 2.3.4. Extraction Recovery and Matrix Effect

The extraction recovery and matrix effect data of the three analytes are summarized in Table 4. The extraction solvent used in the experiment showed good extraction efficiency. The extraction recoveries of QC samples and the I.S. were 81.30%–84.21%, 78.87%–83.89%, 84.53–88.96%, and 94.74%, respectively. The matrix effects were 95.14%–98.35%, 92.56%–99.51%, and 94.53%–100.2% for purpurin, munjistin, and mollugin at three QC levels, respectively, indicating that the impact from the plasma matric was negligible. These results demonstrated that the values were all in the acceptable ranges, confirming that the evaluated method was free of matrix effects.

#### 2.3.5. Stability Experiments

The stability of the three analytes was investigated under the given four conditions. The results in Table 5 indicate that the three analytes are stable in plasma after three freeze-thaw cycles at room temperature for 4 h. The post-preparative stability of the analytes also verified that no significant degradation occurred when the extracted samples were kept at 4 °C for 12 h. Moreover, all the investigated compounds were stable at −20 °C for at least one month.

#### 2.3.6. Application to the Pharmacokinetic Study

**Figure 4:**
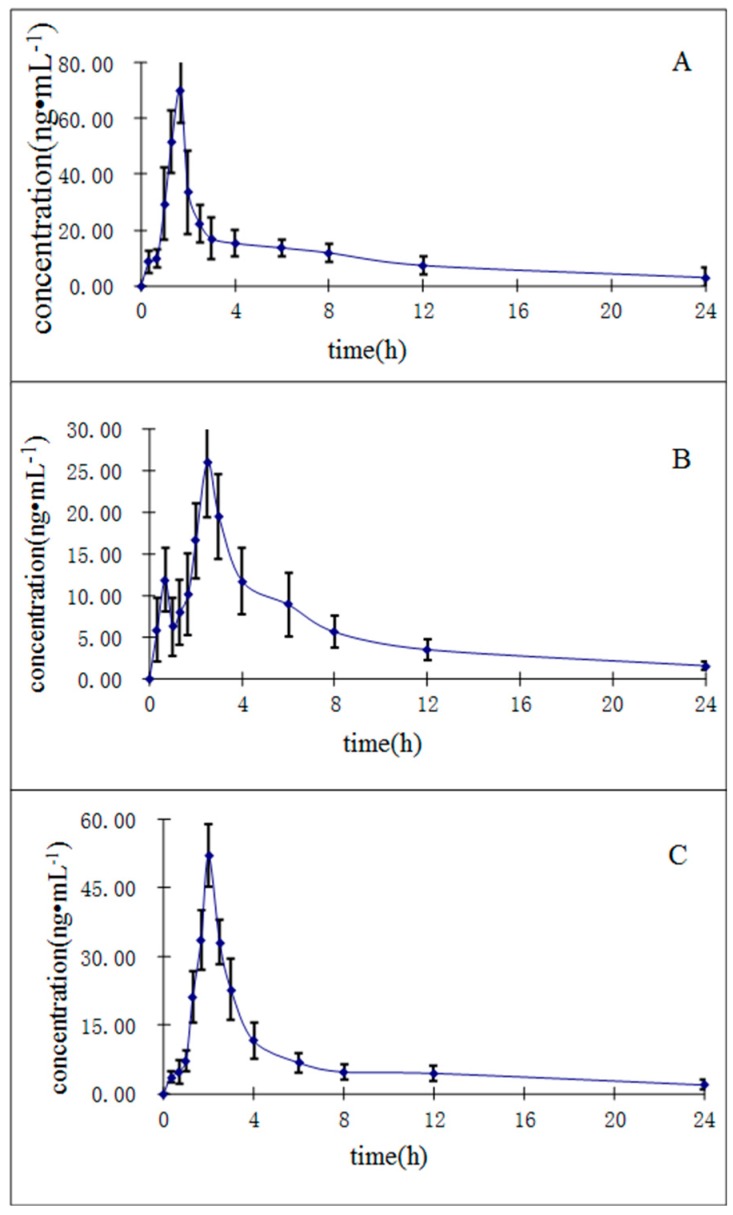
Mean ± SD (*n* = 12) plasma concentration-time profiles of purpurin (**A**); munjistin (**B**); and mollugin (**C**) in rats after oral administration of *R. cordifolia* extract.

The UHPLC-MS/MS method described herein was applied to investigate the pharmacokinetics of the three constituents in *R. cordifolia* after a single oral administration of 0.82 g/kg of extract (equivalent to 6.3 μg/g for mollugin, 1.3 μg/g for munjistin, and 0.68 μg/g for purpurin) to twelve rats. We determined the oral dosage for the rats corresponding to the human dosage recommended in the Chinese Pharmacopoeia (2015 edition). The human dosage of *R. cordifolia* in the Chinese Pharmacopoeia is 10 g/day, so we calculated the final oral dosage as 0.82 g/kg in rats based on the Meeh-Rubn method. The mean plasma concentration-time curves (*n* = 12) of the three analytes are shown in Figure 4.

The relevant pharmacokinetic parameters, including the half-time (*t*_1/2_), maximum plasma concentration (*C*_max_), time to reach the maximum concentration (*T*_max_), and areas under concentration-time curve (AUC_0→t_ and AUC_0→∞_) calculated by non-compartment model, are listed in Table 6. As shown in Table 6, the *C_max_* values are 70.1 ± 11.8, 26.09 ± 6.6, 52.10 ± 6.71 ng/mL for purpurin, munjistin, and mollugin, respectively. These results may be caused by differences in the contents of the three compounds in the *R. cordifolia* extract. The *T*_max_ ranged from 1.61 ± 0.24 min to 2.58 ± 0.19 min, which shows that the absorption of the three compounds *in vivo* is different. The AUC_0→t_ ranged from 110.31 ± 20.10 to 231.56 ± 57.57 ng∙h/mL. These results should support further studies on the pharmacokinetics, pharmacy and toxicity of the *R. cordifolia* and those seeking to determine the efficacy of this TCM in clinical therapeutic research.

## 3. Materials and Methods

### 3.1. Materials and Reagents

Reference standards of purpurin, munjistin, and mollugin were isolated from *R. cordifolia* and identified by our laboratory. On the basis of UV, MS, NMR and HPLC analysis, the chemical structures and the purities (purity ≥ 98%) of isolated reference standards were confirmed. Bifendate (Lots: 100192-200503) was purchased from Chengdu Must Bio-Technology Co., Ltd. (Chengdu, Sichuan, China) and used as an internal standard (I.S.).

The following solvents and chemicals were used in our experiments: J & K Medical (Beijing, China) provided the HPLC-grade methanol (MeOH) and acetonitrile (CH_3_CN). HPLC-grade ammonium acetate was purchased from DIKMA Technologies (Beijing, China). All other reagents were of analytical-grade. Ultrapure water was prepared using a Milli-Q water purification system (Millipore, Molsheim, France).

The dried roots and rhizomes of *R. cordifolia* were obtained from Bozhou (Anhui, China), the largest Chinese herbal medicine distribution center in the world, and were identified by Professor Zhenyue Wang in Heilongjiang University of Chinese Medicine. A voucher specimen (Art. No. 20160001) was deposited in the College of Pharmacy, Harbin Medical University, China.

### 3.2. Instruments and Analytical Conditions

The UHPLC-MS system (Agilent Technologies 1290 series) consisting of a quaternary pump, an automatic degasser, and an auto-sampler, coupled to a 6430 QQQ-MS instrument (Agilent, Santa Clara, CA, USA) with an ESI interface. Chromatographic separation was carried out using a C_18_ column (ACQUITY UPLC^®^ HSS T3, 1.8 μm, 2.1 × 100 mm). The column temperature was set at 40 °C and a 5 μL aliquot of the sample solution was injected for each injection. Chromatography was performed using a gradient system consisting of mobile phase solution A (5 mM ammomium acetate in water) and solution B (methanol) at a flow rate of 0.4 mL/min. The gradient elution method was as follows: 0–6.5 min 90% B; 6.5–8 min linear increase to 98% B; 8.0–8.5 min linear decrease to 90% B. The analysis time was 8.5 min for each injection.

The positive ionization mode was used for compound ionization. An Agilent Mass Hunter workstation was used to control the equipment and for data acquisition and analysis. The quantification was obtained in multiple reaction monitoring (MRM) mode with the precursor-product ion transition at *m*/*z* 274.2→70.1 (88.1) for purpurin, *m*/*z* 284.3→57.0 (71.9) for munjistin, *m*/*z* 285.0→252.9 (183.0) for mollugin, and *m*/*z* 418.9→342.9 (284.8) for bifendate (I.S.), respectively. The fragment and collision energy are shown in Table 1. The product ion scan spectra of the analytes and the I.S. are shown in Figure 2. High-purity nitrogen (N_2_) was used as the nebulizing gas, and nitrogen (N_2_) was used as the drying gas at a flow rate of 12 L/min. The mass spectrometer was operated at a capillary voltage of 4000 V, source temperature of 100 °C and desolvation temperature of 350 °C.

### 3.3. Preparation of R. Cordifolia Extract 

An ultrasonic extraction method was used for the extract preparation. Dried powder (200 g) of *R. cordifolia* was ultrasonically extracted twice (1 h each time) with methanol at a material to liquid ratio of 1:6 and then filtered. The combined filtrate was evaporated to dryness, and the residue was reconstituted in water to a concentration equivalent to 0.1 g/mL of the *R. cordifolia* extract.

### 3.4. Preparation of Calibration Standards and Quality Control (QC) Samples

Standard mixed stock solution of purpurin, munjistin, and mollugin were prepared in methanol at concentrations of 0.264 mg/mL, 0.515 mg/mL and 2.45 mg/mL, and then further diluted in methanol to produce the working solutions with a series of concentrations. The I.S. solution (2000 ng/mL) was obtained by diluting the stock solution in methanol for routine use. The samples for the standard calibration curves were prepared by spiking appropriate amounts of the standard solutions into 100 μL of blank plasma to yield the final calibration concentrations of 0.264, 0.528, 2.11, 5.28, 26.4, 52.8, 264 ng/mL for purpurin, 0.515, 1.03, 4.12, 10.3, 51.5, 103, 515 ng/mL for munjistin, and 2.45, 4.90, 19.6, 49.0, 245, 490, 2450 ng/mL for mollugin, respectively. The QC samples were prepared in drug-free plasma at four different concentration levels, high QC (211/412/1960 ng/mL), medium QC (26.4/51.5/245ng/mL), low QC (0.528/1.03/4.90 ng/mL), and LLOQ (0.264/0.515/2.45 ng/mL), for purpurin, munjistin, and mollugin. All the stock and preparing solutions were maintained in the freezer at 4 °C and brought to room temperature before use.

### 3.5. Animal Experiments

Twelve male Sprague-Dawley (SD) rats (body weight 220 ± 20 g) were obtained from the experimental animal center of Harbin Medical University and adapted to a constant-temperature (25 °C) room. The animal handling procedures were approved by the institutional ethics committee and conformed to the principles of the International Guide for the Care and Use of Laboratory Animals. Before the experiment, the rats were fasted for 12 h and had access to water during the research period. Blood (0.4 mL) was collected from the orbital venous plexus at several specific time points (0, 0.33, 0.67, 1, 1.3, 1.67, 2, 2.5, 3, 4, 6, 8, 12 and 24 h) after the oral administration of *R*. *cordifolia* (0.82 g/kg). After centrifugation at 12000 rpm for 5 min, the plasma was collected immediately and stored at −20 °C until sample analysis.

### 3.6. Plasma Sample Preparation

Before the experiment, the plasma samples were thawed naturally at room temperature. As a liquid-liquid extraction method, 100 μL of methanol and 50 μL of I.S. (2000 ng/mL) were added to a rat plasma sample (100 μL), and then the content was mixed by a vortex mixer for 30 s. After this step, a 3 mL aliquot of ethyl acetate (extraction agent) was accurately added and the mixture vortexed for 60 s. Then the samples were put into a centrifuge. The organic supernatant was pipetted into clean glass tubes individually after centrifugation 4000 rpm for 5 min. The upper organic layer was removed and evaporated to dryness at 40 °C under a stream of nitrogen. The residue was then reconstituted with 100 μL of mobile phase (5 mM ammonium acetate in water/methanol, 10:90, *v*/*v*), vortexed for 30 s and filtered by a 0.22 μm membrane. A 5 μL aliquot of the solution was injected into the UHPLC-MS/MS system for analysis.

### 3.7. Method Validation

The method was fully validated according to the Food and Drug Administration (FDA) guidance for biological method validation (FDA 2001 [28]) in terms of specificity and selectivity, linearity, intra- and inter-day precisions, accuracy, stability, matrix effects, and extraction recovery. The validation runs were conducted on three consecutive days. Each validation run consisted of two sets of calibration standards and six replicates of QC samples at three different concentrations.

#### 3.7.1. Specificity and Selectivity

Selectivity is the ability of an analytical method to differentiate and quantify the analytes in the presence of other components in the sample and endogenous substances in plasma. Comparing and analyzing blank samples from six rats, blank plasma spiked with the three analytes and the I.S., and the plasma sample from rats fed the extract of *R. cordifolia*, there were no detectable interfering peaks at the respective retention times of quantification of the three analytes (LLOQ).

#### 3.7.2. Linearity and Lower Limits of Quantification (LLOQ)

Seven different concentrations of standard plasma samples were used to establish calibration curves on three consecutive days. The linearity of each calibration curve was determined by plotting the peak area ratio (y) of the analyte to the I.S. against the nominal concentration (*x*) of the analyte with weighted (1/*x*^2^) least square linear regression. The LLOQ was determined as the lowest concentration point of the calibration curve at which the measured precision, expressed as the relative standard deviation (RSD), was required to be within ±20%, and the accuracy, expressed as relative error (RE%), was required to be within ±20%.

#### 3.7.3. Precision and Accuracy

Four different concentration levels of QC samples were selected to verify the intra- and inter- day accuracy and precision. Four levels of QC samples (LLOQ, LQC, MQC, and HQC) in six replicates were analyzed on the same day to determine the intra-day precision and on three consecutive days to evaluate the inter-day precision. The precision was expressed as the RSD with a criterion of less than 15% and the accuracy was assessed by comparing the measured concentration with its nominal value calculated as the RE with requirement of within ±15%.

#### 3.7.4. Extraction Recovery and Matrix Effect

The extraction efficiency of the three analytes were determined by analyzing six replicates of the plasma samples at their respective LQCs, MQCs and HQCs. To investigate recovery and matrix effect, extracted samples (A), unextracted samples (B), and post-extracted spiked samples (C) were analyzed in the same assay. The extraction recovery was expressed as (mean observed peak areas of A)/(peak areas of C) × 100% and the matrix effect was acquired by calculating (mean observed peak areas of A)/(peak areas of B) × 100%. The extraction recovery and the matrix effect were similarly evaluated for the I.S. at one concentration.

#### 3.7.5. Stability Experiments

To study the stability, QC samples of the three analytes at three QC levels (LQC, MQC, and HQC) were added into drug-free plasma samples and each levels contained six replicates. Four different manipulations were designed to assess the stability involving short-term stability (storage for 4 h at ambient temperature), long-term stability (storage for 2 weeks at −20 °C), freeze-thaw stability (three freeze at −20 °C and thaw cycles), and post-preparative stability (storage for 12 h after sample preparation at 4 °C). A series of concentrations of stability testing QC samples was obtained by the calibration curve which was established from freshly prepared standard samples.

### 3.8. Application to Pharmacokinetic Study

Noncompartmental methods were applied to calculate the following pharmacokinetic parameters. The peak plasma concentration (*C*_max_) and the time of maximum plasma concentration (*T*_max_) were observed directly from the measured data. The elimination rate constant (*K_e_*) was calculated using the linear regression of the terminal points in a semi-log plot of the plasma concentration against time. The elimination half-life (*t*_1/2_) was calculated using the formula:

*t*_1/2_ = 0.693/*K_e_*

The area under the peak plasma concentration-time curve (AUC_0→t_) to the last measurable plasma concentration (*C_t_*) was estimated using the linear trapezoidal rule. The area under the plasma concentration-time curve to time infinity (AUC_0→∞_) was calculated as following:

AUC_0→∞_ = AUC_0-t_ + *C_t_*/*K_e_*

## 4. Conclusions

A specific, simple and sensitive UHPLC-MS/MS method has been developed and validated for the quantification of purpurin, munjistin, and mollugin in rat plasma using liquid-liquid extraction as sample preparation procedure for the first time. This validated method was found to be precise, fast and highly accurate, and it met all bio-analysis requirements. The method has been applied to the pharmacokinetic study of purpurin, munjistin, and mollugin in rat plasma. It is expected that the results of this study will provide some useful references to the further pharmacological study of *R. cordifolia*.

## Figures and Tables

**Table 1:** Optimized mass spectrometric parameters for the LC/MS analysis of purpurin, munjistin, mollugin and bifendate in MRM mode.

Analyte	Precursor Ion, *m*/*z*	Product Ion, *m*/*z*	Qualifier Ion, *m*/*z*	Fragment, V	Collision Energy, V	Cell Accelerator Voltage, V	Polarity
Purpurin	274.1	70.1	88.1	151	31	5	positive
Munjistin	284.3	57.0	71.9	156	32	5	positive
Mollugin	285.0	252.9	224.8	101	8	5	positive
Bifendate	418.9	342.8	284.8	78	18	5	positive

**Table 2:** The regression equations, linear ranges and LLOQs for the determination of the analytes in rat plasma.

Compounds	Regression Equation	*R*^2^	Linear Range (ng/mL)	LLOQ (ng/mL)
Purpurin	*Y* = 3.458 × 10^3^*X* + 12.73	0.9927	0.264–264	0.264
Munjistin	*Y* = 3.067 × 10^3^*X* + 4.827	0.9979	0.515–515	0.515
Mollugin	*Y* = 9.599*X* + 4.826	0.9939	2.45–2450	2.45

**Table 3:** Precision and accuracy of the determination of purpurin, munjistin, and mollugin in rat plasma (*n* = 18, 6 replicates per day for 3 days).

Compounds	Spiked Concentration (ng/mL)	Measured Concentration (ng/mL)	Accuracy (%)	Intra-Day Precision (%)	Inter-Day Precision (%)
Purpurin	0.264	0.256 ± 0.02	−2.9	6.0	14.2
	0.528	0.50 ± 0.07	−5.10	14.00	12.09
	26.4	24.5 ± 3.10	−7.18	12.96	10.09
	211	242.3 ± 24.3	14.75	10.54	5.07
Munjistin	0.515	0.503 ± 0.06	−2.3	12.6	7.3
	1.03	1.17 ± 0.11	13.88	8.75	11.94
	51.5	55.57 ± 2.67	7.89	4.88	4.21
	412	417.1 ± 37.2	1.24	8.43	12.10
Mollugin	2.45	2.58 ± 0.32	5.2	13.0	7.6
	4.90	5.35 ± 0.66	9.22	12.33	12.30
	245	261.7 ± 38.1	6.82	14.84	12.51
	1960	1684 ± 211	−14.05	13.15	6.61

**Table 4:** Matrix effects and extraction recovery for the analytes in rat plasma (*n* = 6).

Compounds	Spiked Concentration (ng/mL)	Matrix Effect		Extraction Recovery	
		Mean (%)	RSD (%)	Mean (%)	RSD (%)
Purpurin	0.528	98.35	4.182	84.21	9.143
	26.40	95.14	5.012	81.30	5.667
	211.0	95.43	7.447	82.63	3.884
Munjistin	1.030	93.74	2.720	78.87	6.317
	51.50	92.56	9.851	83.89	6.879
	412.0	99.51	4.619	83.46	2.838
Mollugin	4.900	98.85	5.103	84.53	8.346
	245.0	100.2	3.534	85.90	5.074
	1960	94.53	7.476	88.96	4.090
I.S.	2000	96.03	4.225	94.74	11.91

**Table 5:** Stabilities of the analytes in rat plasma (*n* = 6).

Analytes	Spiked Concentration (ng/mL)	Stability (% RE ^a^)			
		Three Freeze-Thaw	Short-Term	Long-Term	Post-Preparative
Purpurin	0.528	1.92	−13.38	−2.59	−14.02
	26.4	−5.35	−5.35	−2.68	−2.00
	211	14.76	14.76	12.08	13.68
Munjistin	1.03	9.04	11.56	13.97	11.15
	51.5	9.46	6.73	5.56	7.04
	412	−2.82	7.31	−0.77	−10.15
Mollugin	4.90	10.26	9.90	4.57	11.15
	245	8.78	−4.35	−0.17	0.65
	1960	−13.17	−8.69	−8.55	14.38

^a^ RE is expressed as (measured concentration/freshly prepared concentration^−1^) × 100%.

**Table 6:** Pharmacokinetic parameters of the three constituents in rats after oral administration of *R. cordifolia* extract. (mean ± SD, *n* = 12).

Compounds	*C*_max_ (ng/mL)	*T*_max_ (h)	*t*_1/2_ (h)	AUC_0→t_ (ng∙h/mL)	AUC_0→∞_ (ng∙h/mL)
Purpurin	70.10 ± 11.78	1.61 ± 0.24	9.52 ± 2.68	205.90 ± 36.12	243.16 ± 35.91
Munjistin	26.09 ± 6.59	2.58 ± 0.19	9.22 ± 2.75	110.31 ± 20.10	136.34 ± 21.19
Mollugin	52.10 ± 6.71	1.99 ± 0.21	9.02 ± 2.14	231.56 ± 57.57	296.49 ± 40.88
